# Comparative Analysis of *Begonia* Plastid Genomes and Their Utility for Species-Level Phylogenetics

**DOI:** 10.1371/journal.pone.0153248

**Published:** 2016-04-08

**Authors:** Nicola Harrison, Richard J. Harrison, Catherine A. Kidner

**Affiliations:** 1 NIAB EMR, East Malling, Kent, United Kingdom; 2 The University of Reading, Whiteknights, Reading, Berkshire, United Kingdom; 3 Royal Botanic Gardens Edinburgh, Edinburgh, Scotland, United Kingdom; 4 The University of Edinburgh, Darwin Building, King's Buildings, Edinburgh, Scotland, United Kingdom; University of Western Sydney, AUSTRALIA

## Abstract

Recent, rapid radiations make species-level phylogenetics difficult to resolve. We used a multiplexed, high-throughput sequencing approach to identify informative genomic regions to resolve phylogenetic relationships at low taxonomic levels in *Begonia* from a survey of sixteen species. A long-range PCR method was used to generate draft plastid genomes to provide a strong phylogenetic backbone, identify fast evolving regions and provide informative molecular markers for species-level phylogenetic studies in *Begonia*.

## Introduction

**Fig 1:**
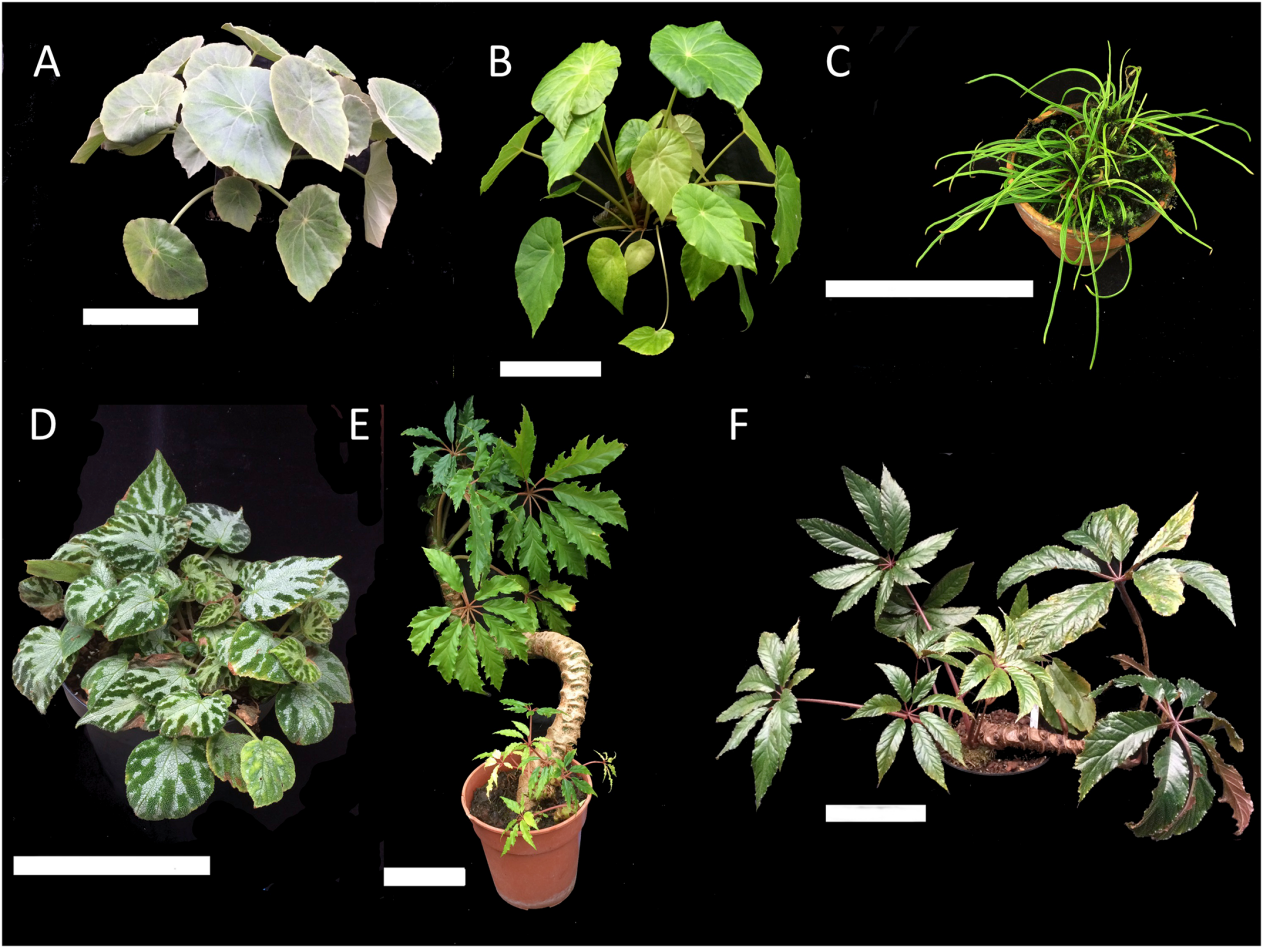
A selection of *Begonia* species from this study illustrating the large variation seen in *Begonia* morphology. A: *B*. *peltata* B: *B*. *nelumbiifolia* C: *B*. *bogneri* D: *B*. *involucrata* E: *B*. *carolineifolia* F: *B*. *theimei*. White scale bar is 15cm long.

*Begonia* is one of the most species-rich angiosperm genera with c.1900 pantropically distributed species currently identified [1]. Although *Begonia* species are typical of wet rainforest herbs, the genus also exhibits substantial diversity in ecology, with ranges from dry desert scrub through to wet rainforest, and at altitudes from sea level to over 3000 metres [2]. *Begonia* also shows wide variations of form between closely related species (Fig 1). High speciation rates may be related to limited seed dispersal mechanisms and low level of gene flow in fragmented populations [3–6]. The large numbers of species, pantropical distribution and a solid horticultural background make *Begonia* an excellent system for the study of plant evolution in tropical environments [7].

Several phylogenetic studies have been published for the Begoniaceae [8–17]. Although these studies provide excellent resolution of sectional variation, within some clades resolution to the species level has proved recalcitrant.

The Begoniaceae are placed within Cucurbitales along with six other plant families; Cucurbitaceae, Datiscaceae, Tetramelaceae, Anisophylleaceae, Coriariaceae and Corynocarpaceae [18]. Begoniaceae-like traits are thought to have evolved between 69–46 million years ago (Ma), when the Begoniaceae crown group diverged (Goodall-Copestake 2005). A geographic origin for this family has proven elusive owing to the lack of fossil evidence, although Clement *et al*, who investigated the ancestral relationship between the two genera of Begoniaceae; *Begonia* and *Hillebrandia*, have suggested that a boreotropic (Northern hemisphere during the Eocene epoch) or a Malaysian-Pacific origin is most likely [10].

Begoniaceae phylogenetic studies all indicate that the most basal *Begonia* species are African, from which both Asian and American *Begonia* species are derived [11]. The arrival of Begonias on the South American continent is estimated approximately 10–12 My [15–17]. Speciation patterns in South America show relatively recent radiations, which are difficult to resolve with traditional, angiosperm molecular markers (such as ITS, *TrnL*, *MatK*). This has led to the need to develop new, informative *Begonia*-specific markers.

In this study, we focussed on the American *Begonia* group, Section *Gireoudia*, the largest of the sixteen American sections, which are predicted to have undergone rapid radiation during or just after the closure of the isthmus of Panama (15,18). Moonlight *et al* resolve the section level patterns of phylogenetic diversity in south American *Begonia* by the use of three non-coding plastid regions (*ndhA* intron, *ndhF-rpl32* spacer and *rpl32-trnL* spacer) however there was no robust resolution at species level; this limits their ability to resolve the species level patterns critical for understanding biogeographic structure [15].

In this study, we used high-throughput sequencing to address the problem of poor resolution in this group of *Begonia* species. The aim was to generate complete plastid genomes for a number of Gireoudia species with outgroups from the rest of the genus. Comparative sequence analyses were then be used to identify suitable molecular markers for low-level phylogenetic studies in *Begonia*.

Since the first plastid genome was sequenced, that of *Nicotiana tabacum*, [19], there have been a total of 826 whole plastid genome sequences submitted to Genbank (as of September 2015, using search terms ‘chloroplast’ and ‘plastid’). This data provides an overview of structural and sequence diversity amongst angiosperms. The typical plastid genome is circular in structure with a standard size of between 120–160 kb, and generally containing a quadripartite configuration [20,21]. These four components comprise; the large single copy (LSC), the small single copy (SSC) and two inverted repeats (IRa and IRb). Most plant plastid genomes have all these features, however there are a few lineages that differ. One well-studied example is within the legumes where one clade has lost one of the inverted repeats, this group is referred to as the inverted repeat-lacking clade (IRLC), [22,23]. Other losses from plastid genomes can be seen in the gymnosperms where it has been found that conifers have also lost an inverted repeat and in addition, have undergone structural changes compared to angiosperms [24]. Plastome size can also be variable within lineages. *Pelargonium x hortorum* has a plastid genome of 217.9 kb, 40% bigger than typical plastid genomes and is specific to the genus. The large size is due to extreme expansion in the size of the inverted repeats, where the size of each IR is 75.7 kb, this is in contrast to the average IR size for angiosperms which is normally between 10–30 kb [25].

## Materials and Methods

### Taxon sampling

A total of sixteen species were chosen for plastid genome sequencing: nine species to represent interspecific variation from within sect. *Gireoudia*: *B*. *conchifolia*, *B*. *plebeja*, *B*. *carolineifolia*, *B*. *stigmosa*, *B*. *peltata*, *B*. *nelumbiifolia*, *B*. *theimei*, *B*. *sericoneura*, *B*. *involucrata*; two species to represent additional American sections: *B*. *pustulata* (sect. *Weilbachia)*, *B*. *solananthera* (sect. *solananthera)*; two Asian and three African species: *B*. *venusta* (sect. *Platycentrum*), *B*. *varipeltata* (sect. *Petermania*) and *B*. *dregei* (sect. *Augustia)*, *B*. *socotrana* (sect. *Peltaugustia*), *B*. *bogneri* (sect. *Erminea*), respectively. The list of taxa used along with the sources of plant material and accession numbers are provided in Table 1. The sectional and geographical affinities of the taxa are also described with Sectional placements following Doorenbos et al, (1998), [26].

**Table 1:** List of *Begonia* taxa used in this manuscript accompanied by the country of origin, source of plant material and accompanying accession numbers. All plant material was sampled from the living collections at the Royal Botanic Gardens Edinburgh (RBGE) or Glasgow Botanic Gardens (GBG).

Species	Section	Region	Accession	Institution
*B*. *plebeja*	*Gireoudia*	America	20051406	RBGE
*B*. *conchifolia*	*Gireoudia*	America	20042082	RBGE
*B*. *stigmosa*	*Gireoudia*	America	20051413	RBGE
*B*. *peltata*	*Gireoudia*	America	2004078	RBGE
*B*. *nelumbiifolia*	*Gireoudia*	America	19791880	RBGE
*B*. *theimei*	*Gireoudia*	America	20042079	RBGE
*B*. *sericoneura*	*Gireoudia*	America	GL00400185	GBG
*B*. *involucrata*	*Gireoudia*	America	GL00410057	GBG
*B*. *pustulata*	*Weilbachia*	America	GL02212482	GBG
*B*. *carolineifolia*	*Gireoudia*	America	GL00108299	GBG
*B*. *solananthera*	*Solananthera*	America	19991101	RBGE
*B*. *bogneri*	*Erminea*	Madagascar	19860844	RBGE
*B*. *venusta*	*Platycentrum*	Asia	20021596	RBGE
*B*. *varipeltata*	*Petermania*	Asia	20040641	RBGE
*B*. *dregei*	*Augustia*	Africa, South	20000905	RBGE
*B*. *socotrana*	*Peltaugustia*	Africa, Socotra	20000325	RBGE

### DNA extraction

Silica dried plant material (approximately 20mg) was disrupted using a Qiagen Tissuelyser system, then total genomic DNA was extracted using Qiagen DNeasy Tissue Kit (Cat No. 69504) following manufacturers recommendations. Total genomic DNA was eluted in 2 x 50μl sterile distilled water and stored at -20°C.

### Long-range PCR (LR-PCR) primers

The lack of an available reference *Begonia* plastid genome led to the development of primers based on conserved angiosperm plastid sequences [27]. Chung *et al* (2007), designed primers from regions that were conserved between three distantly related plant species (*Arabidopsis thaliana*, *Spinacia oleracea* and *Nicotiana tabacum*) by analysing available whole plastid genomes, and designing their primers to have an amplicon size of 3 kb. In this study, we reshuffled the conserved plastid primers to make new primer combinations in order to produce 10 kb amplicons with 500–1000 bp overlaps between amplicons. The large overlap was designed to reduce the types of errors encountered by Cronn *et al* (2008) during sequence assemblies, such as complementary forward and reverse primers at a single site precluding them from obtaining genomic sequence for those positions, [28] Eighteen primers pairs were initially designed and tested to amplify plastid regions in *Begonia* species, (S1 Table). For each *Begonia* accession, 18 PCRs were performed in separate reactions and pooled. In some cases amplification was poor and additional primer pairs were developed and substituted, (S1 Table).

### Long-range Polymerase Chain Reaction (LR-PCR)

LR-PCR was carried out in 25μl reactions containing: approx 20ng genomic DNA, 1x LongAmp PCR buffer (New England Biolabs, M0323G), 300 μM dNTP, 0.4 μM of each primer, 2.5 units of LongAmp *Taq* DNA polymerase, Nuclease-free water to 25 μl. PCR amplification involved an initial denaturing step of 94°C for 30 secs, then 94°C for 10 secs, 50°C for 30 secs, 65°C for 9 minutes for 45 cycles, and a final extension period of 65°C for 10 mins followed by a 4°C hold. All reactions were carried out in a MJ Research PTC100 thermocycler.

### Verification of plastid amplification

To verify the long-range PCRs had amplified plastid sequences, the amplicons generated from *B*. *nelumbiifolia* were pooled and the pooled sample was cloned using standard TOPO^®^ cloning vectors and protocols. Twenty-four random clones were sequenced using traditional Sanger sequencing by ‘The Genepool’ sequencing facility, Edinburgh. The sequencing results were subject to BLAST searches against the non-redundant database in GenBank using megablast parameters for highly similar sequences, (http://www.ncbi.nlm.nih.gov/). In cases where no sequence match was found, BLAST stringency was reduced to blastn default algorithm parameter selection for similar sequences.

### Illumina plastid genome sequencing

Each LR-PCR pool was converted into barcoded, paired-end Illumina sequence libraries for the Illumina GAIIx platform, which were further pooled to create multiplexed sequencing libraries using standard illumina chemistry and protocols. Two Illumina GAIIx lanes were used, each with a multiplex library containing eight barcoded samples. Illumina sequencing consisted of the generation of 50 bp paired-end reads with an insert size of approximately 250bp on an Illumina GAIIx platform. Sequencing was performed by ‘The Genepool’ sequencing facility, Edinburgh, UK (http://genomics.ed.ac.uk).

### Plastid genome assembly

Using an Illumina GAIIx sequencing system, a total of 6.2 Gb of data were generated. Reads were deconvoluted using the barcodes into individual accessions and the barcodes removed. All accessions were subject to *de novo* assembly using Velvet Software v.1.2.03 [29]. Assembly parameters were optimized by performing several assemblies, using a range of Kmer sizes. The results of the assemblies were assessed through the assembly statistics, (i.e. N50 statistics) alongside BLAST analysis of contig sequences. Final parameters used: kmer length 31; expected coverage 1000; coverage cutoff 5; minimum contig length 100bp; -shortpaired; insert length 200. The following settings were used during compilation of velvet software: CATEGORIES = 2; MAXKMERLENGTH = 65; OPENMP; LONGSEQUENCES; BIGASSEMBLY. *Begonia de novo* contigs were verified using Blastn against the non-redundant database in GenBank using megablast and default parameters (http://www.ncbi.nlm.nih.gov/).

### *Begonia peltata* draft plastid genome

The draft sequence of *B*. *peltata* was determined by aligning the *B*. *peltata* assembled contigs to the *Cucumis sativus* plastid genome [GenBank accession: DQ865976.1] using the reference-guided assembler Maqview(1) (http://maq.sourceforge.net/maqview-man.shtml).

### Gap-closing in the draft *B*. *peltata* plastid genome

Custom primers (S2 Table) were designed—based on sequence from the draft *B*. *pelata* plastid genome—to confirm the junctions of the inverted repeats (IR) and to close seven gaps where the contigs did not join in the *B*. *peltata* draft plastid genome. The products from successful PCR amplifications were Sanger sequenced and used to improve the *B*. *peltata* plastid genome.

### Multiple genome alignment of *Begonia* plastid genomes

The draft sequence generated for *B*. *peltata* was used as a reference to map each set of assembled contigs for all sixteen *Begonia* species (including *B*. *peltata*). Plastid genome contigs were aligned to *B*. *peltata* using MAFFT v6.717 (Multiple Alignment using Fast Fourier Transform) [30] applying the iterative refinement method (FFT-NS-i) and using default parameter settings (gap opening penality: 1.53, offset-value: 0.0) and then visually inspected and manually adjusted in the software program Geneious Pro 5.6.3 (http://www.geneious.com/).

### Modeltest and maximum likelihood analysis

The program Modeltest [31] was used to test models of evolution on the plastid genome alignment for maximum likelihood analyses. The molecular substitution model chosen for the plastid genome alignment was GTR+I+G as selected by the Akaike’s Information Criterion (AIC).

### MrModelTest and Bayesian analysis

The program MrModeltest 2.2 [32] was used to test models of evolution on the plastid genome alignment for Bayesian Inference analyses. The molecular substitution model chosen for the plastid genome alignment was GTR+I+G selected by the AIC.

### Maximum parsimony

The aligned plastid DNA matrix for sixteen *Begonia* species was subjected to maximum parsimony (MP) analysis in PAUP 4.0b10 [33]. A heuristic search strategy of 10,000 random sequence addition replicates with tree bisection-reconnection (TBR) branch swapping, saving 10 trees per replicate, with MULTREES on, steepest descent off, was implemented. A bootstrap analysis [34] was performed to evaluate the robustness of clades using a fast stepwise addition search algorithm with 1000 replicates. A 50% majority-rule consensus tree was calculated from all the most parsimonious trees.

### Maximum likelihood

Maximum likelihood (ML) analysis was implemented using PhyML [35] with GTR+I+G and determination of a 50% majority rule consensus tree from 1000 bootstraps. The appropriate substitution model was selected for the dataset using Modeltest [31] based on AIC and hierarchical Likelihood Ratio Test (hLRT).

### Bayesian inference

Bayesian inference (BI) analyses were performed in MrBayes v3.1.2 [36] on unordered and equally weighted characters with the following settings. The evolutionary model employed six substitution types (“nst = 6”), with base frequencies set to the empirically observed values (“basefreq = empirical”). Rate variation across sites was modeled using a gamma distribution (“rates = invgamma”). The Markov chain Monte Carlo search was run with 4 chains for 1,100,000 generations, with trees sampled every 200 generations and the first 1000 trees discarded as ‘burn-in’. Phylogenetic trees were visualized using Figtree [37].

### Support values

For the BI analyses, a 90% posterior probability (PP) lower threshold was considered to indicate moderate support and a 95% lower threshold to indicate well supported relationships. For the MP and ML analyses, a 70% bootstrap support value lower threshold was considered to indicate moderate support, and an 85% lower threshold to indicate well-supported relationships.

### A comparison of variation between the LSC, SSC and IR regions

The *Begonia* plastid alignment was partitioned into the small single copy (SSC), large single copy (LSC) and inverted repeat (IR) regions after consideration of the common boundaries [38,39] and visualization of the discrepancies found in the alignment itself (discrepancies seemed to be a contained in the regions predicted to span the junctions of the inverted repeats). Each region was subjected to phylogenetic analyses along with descriptive statistics produced using Geneious software, as well as manually calculated. In addition, descriptive statistics were produced only for species in sect. *Gireoudia* for each region to look at variation within sect. *Gireoudia*.

### Selection of phylogenetically informative regions for low-level studies in *Begonia*

A sliding windows analysis was performed on the large-scale Begonia alignment in the commercial software, Geneious. A window size of 10 bp was used to assess mean pairwise identity over all pairs in the column across the whole alignment. The sliding windows analysis was used to identify regions of the alignment that contain potential phylogenetically informative regions. These regions were then visually inspected to ensure that there was as little missing data as possible and that unambiguously aligned regions were not included in the final analyses. For each new region identified, the sequence alignment was subjected to a maximum likelihood analysis using PhyML with the following evolutionary model, GTR+I+G and determination of a 50% majority rule consensus tree from 1000 bootstrap replicates. The resulting phylogenetic trees were visually compared with the phylogenetic tree from the whole plastid genome alignment, particularly with respect to good bootstrap support and the grouping of the American *Begonias*. A small selection of the potentially phylogenetically informative regions identified through ML analyses were subjected to further computationally intensive phylogenetic analyses using Bayesian Inference performed in MrBayes v3.1. The phylogenetic trees produced were again compared to the phylogenetic tree from the large-scale plastid genome alignment for similarity and statistical support. Blast searches were performed on the consensus sequence for each alignment to determine/confirm sequence identity.

### Plastid genome annotation

Plastid genome annotation was performed using DOGMA [40] and putative annotations were confirmed using blast sequence similarity search.

Plastid assemblies and alignments are available in Dryad (doi:10.5061/dryad.cp4mb) and raw reads are in the European Nucleotide Archive, Project PRJEB11898.

## Results and Discussion

Recent and extensive speciation in *Begonia* requires informative markers for full phylogenetic resolution. To identify optimum plastid markers we used a comparison of conserved sequences from angiosperm plastid genomes [27] to design 18 pairs of primers for the generation of 10 kb amplicons with overlapping regions to sequence 16 *Begonia* plastid genomes.

### Plastid genome assembly and features

A total number of 32,611,570 sequence reads were generated, providing 6.2 Gb of data, consisting of 50bp paired-end illumina reads. Expected coverage was determined based on the size of the *Cucumis sativas* plastid genome and ranged from 382–832 (S3 Table). The reads were assembled *de novo* and the number of assembled contigs per genome ranged from 132–679 (Table 2). The contigs were validated by blast searches of the NCBI database. From the sixteen plastid genome datasets, *B*. *peltata* was chosen to create a draft genome as initial assembly statistics indicated that this dataset was the most complete and had the best assembly scores with a total of 310 contigs and an N50 of 9, of which 50 contigs were greater than one kb.

**Table 2:** A summary of the assembly statistics for *Begonia* plastid genome sequencing and assembly.

*Begonia* species	N50	Max contig size	Number of bases in contigs	Number of contigs	Number of contigs > = 1kb	Number of contigs in N50	Number of bases in contigs > = 1kb	GC Content of contigs
*B*. *plebeja*	2918	47014	304482	404	52	12	215845	38.15
*B*. *conchifolia*	10499	44654	218044	153	38	4	179038	38.63
*B*. *stigmosa*	2007	53538	484905	679	101	48	321804	38.69
*B*. *peltata*	5239	51277	302780	310	50	9	233185	37.52
*B*. *nelumbiifolia*	2662	47678	316607	422	56	13	228205	38.17
*B*. *theimei*	2817	41362	297718	312	60	16	224691	37.3
*B*. *sericoneura*	5468	100815	297767	272	46	6	229959	37.78
*B*. *involucrata*	2420	101271	327063	443	54	14	226289	37.41
*B*. *pustulata*	3156	20519	157899	132	40	12	126468	41.02
*B*. *carolineifolia*	8074	53923	296506	175	30	5	161466	37.26
*B*. *solananthera*	1448	17557	251467	352	60	39	152222	43.73
*B*. *bogneri*	3647	28890	216917	185	45	12	169266	38.73
*B*. *venusta*	2919	56049	230527	239	41	8	171398	37.22
*B*. *varipeltata*	4995	26542	263018	266	42	10	205585	37.22
*B*. *dregei*	17989	44260	196321	215	22	3	143794	36.99
*B*. *socotrana*	7363	63731	249706	180	39	5	205824	37.76

The draft *B*. *peltata* plastid genome has a typical angiosperm quadripartite structure. A large single copy (LSC) region (84,812 bp) flanked either side by a pair of inverted repeats, termed IRa and IRb, (predicted to be 26,456 bp) and circularized by a small single copy (SSC) region (16,152 bp). The IR region was predicted from the site of contig termination (the start of sequence duplication) in the *ycf1* gene in the SSC region, through to contig termination in the *rps19* gene in the LSC. The final draft plastid genome for *B*. *peltata* covered a total of 127,420 bp excluding the IRb.

The *B*. *peltata* genome was made more complete by the development of custom primers to aid with contig orientation and gap-closing. A total of five out of seven gaps were closed using traditional Sanger sequencing. Sequence analysis revealed that these gaps were present because of low complexity and/or homopolymer runs These events are common when assembling sequences *de novo* with Illumina short read data, as an increase in identical nucleotides or tandem repeats reduces the confidence of the assembly and can result in contig termination [29]. Two gaps were located at the predicted junctions of the IRb region. One end of the IRb is is predicted to reside within the linear gene grouping of *rpl2-rps19-rpl22* genes. The contig representing this region in the LSC terminates in the *rps19* gene, making this the most likely position for the junction. The *rps19* gene is one of the most common sites for the LSC and IRb junction reported in the angiosperms [39]. The *rps19* gene in *B*. *pletata* is, however, not complete. A megablast search of the non-redundant database using *the B*. *peltata rps19* gene region as the query sequence indicates there is potentially 15 bp missing from the end of the gene sequence in this species, and *rpl22* is missing altogether. We were unable to verify the size and/or presence of these genes due to PCR failure in for all *Begonia* species used in this study. It is possible that these genes are missing or significantly changed in *Begonia*. Therefore, when calculating the total plastome size these genes are estimates based upon those of *Cucumis sativus*. The total length of the complete *B*. *peltata* plastid genome is predicted to be 153,876 bp.

### Genome annotation

**Fig 2:**
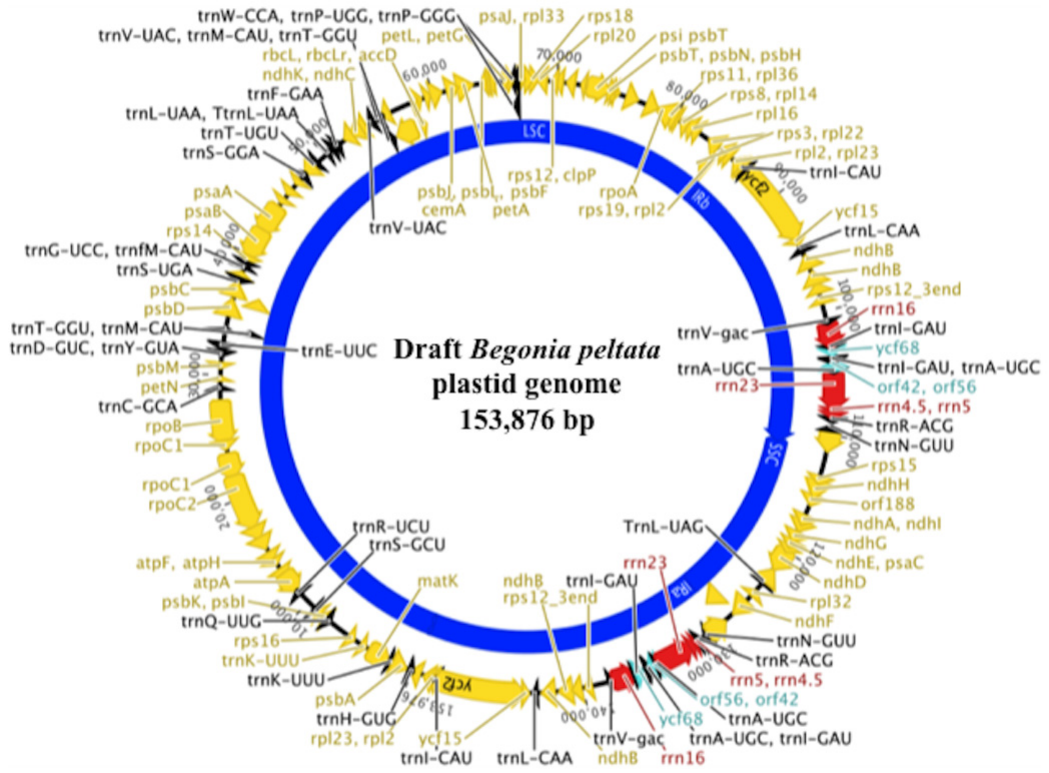
The gene map for the draft *Begonia peltata* plastid genome. The plastid genome size of *B*. *peltata* is predicted to be 153,876 bp in length.

Annotation of the draft plastid genome from *B*. *peltata* predicts 103 genes, including 45 tRNA genes, eight ribosomal RNA genes and six predicted open reading frames (ORFs), (Fig 2). The LSC contains 70 genes and 26 tRNA genes, whilst the SSC contains 13 genes and one tRNA. The predicted inverted repeats each contain ten genes and nine tRNA genes along with four rRNAs and three predicted ORFs (*ycf68*, *orf42* and *orf56*). These results fit well with previously annotated angiosperm plastid genomes, and are highly syntenic with known plastid genomes in the Cucurbitales [27].

### Base composition and codon usage

The nucleotide base composition of the plastid genome of *B*. *peltata* is highly skewed (64.2%), in favour of adenine (A) and thymine (T) bases, whilst poorly represented in cytosine (C) and guanine (G) bases (35.8%). This unequal balance is typical of plastid genomes throughout angiosperm lineages with average AT base composition reported to be around 62% [41], and an AT-rich base composition is also seen in more primitive organisms such as the green algae, *Oedogonium cardiacum* at 70.5% [42]. Theories speculating on the reasoning for high AT-content include the mis-incorporation of A and T bases during replication, a bias in DNA repair machinery and DNA damage through high levels of reactive, and potentially mutagenic species generated during the electron transfer reactions of photosynthesis [43]. The high AT-content is also a general feature of mitochondrial genomes, another endosymbiont with a circular genome.

### Comparison with *Cucumis sativus*

*Cucumis sativus* was the closest relative to *Begonia* whose plastid genome had been sequenced [27]. A sequence alignment between *C*. *sativus* and *B*. *peltata* reveals that the genes are highly syntenic, which is reflective of the general conserved order of plastid genes across land plants [21], however a pairwise identity of 50.1% at the nucleotide level reflects the sequence diversity between these distantly related species. The gene content of both plastid genomes are almost identical except for the loss of the *infA* gene (which codes for the translation initiation factor 1) in *B*. *peltata*. Unfortunately, the *infA* gene is usually found between the genes *rpl36* and *rps8*, this region is also the same region that is missing from all the sequenced *Begonia* plastid genomes in this study and this region is positioned closely (~4.3 Kb) to the genes *rpl22* and *rpl19* discussed previously. Additional plastid sequence obtained as part of the *B*. *conchifolia* genome sequencing project (Kidner *et al*, Unpublished) revealed *infA* has become a pseudogene containing 11 stop codons. The functional loss of the *infA* gene is not unexpected, as this has been previously reported for many angiosperm lineages including *Begonia*, [44]. Millen *et al* (2001) predict a total of four independent losses of *infA* in the Rosids—including the genus *Begonia* although they did not specify which species—with additional separate losses in 24 angiosperm lineages. It is clear that the *infA* gene is one of the least conserved genes in the plastid genome.

### A comparison of variation between the LSC, SSC and IR regions

The analysis of the SSC, LSC and the IR regions show that in the *Begonia* species sampled, the SSC has evolved almost twice as fast as the LSC, and at five times the rate of the IR, Table 3. The variability present in sect. *Gireoudia* only, reflects the same results, Table 4. Many previous phylogenetic studies in *Begonia* have concentrated on molecular markers found in the LSC; *matK* gene [45], *rbcL* gene (10), *trnL-UAA* intron [46], *trnL*–*F* spacer [45], although some of these are protein-coding and will therefore be expected to have a slower evolutionary rate. Recently, Thomas *et al*, (2011) successfully demonstrated the phylogenetic utility of a selection of sequential non-coding regions found in the SSC (*ndhA* intron, *ndhF*–*rpl32* spacer, *rpl32–trnL* spacer) for low-level studies of Asian *Begonia*. These three regions combined (a total alignment length of 4059 bp) were able to determine the species level resolution for several Asian *Begonia* sections [13]. Analysis of the IR region reveals a low substitution rate, confirming the findings of Wolfe *et al*, (1987) who reported that the inverted repeats were more highly conserved than the SSC and the LSC regions. Although their analysis was between highly divergent monocotyledon and eudicot plants, it can be concluded from these analyses that it holds true for species-level comparisons in *Begonia* [47].

**Table 3:** A comparison of the SSC, LSC and the IR regions in *Begonia*.

	All Begonia—16 taxa				Section Gireoudia Only—9 taxa			
	Whole CP	LSC	SSC	IR	Whole CP	LSC	SSC	IR
Alignment Length (bp)	150,255	86,676	18,377	23,222	150,255	86,676	18,377	23,222
Parsimony-uninformative characters (bp)	4804	3295	872	292	1394	992	267	69
Parsimony-informative characters (bp)	1913	1320	447	80	954	643	235	40
Parsimony-uninformative characters (%)	3.20	3.80	4.75	1.26	0.93	1.14	1.45	0.30
Parsimony-informative characters (%)	1.27	1.52	2.43	0.34	0.63	0.74	1.28	0.17
Total % variability	4.47	5.32	7.18	1.60	1.56	1.89	2.73	0.47

**Table 4:** A comparison of DNA sequence alignments for R21, R24 and the *TrnL* regions in *Begonia*.

	All Begonia sp—16 taxa		Sect. Gireoudia Only—9 taxa		
	R21 (SSC)	R24 (LSC)	R21 (SSC)	R24 (LSC)	TrnL-F (LSC)
Alignment Length (bp)	723	1360	723	1360	537
Parsimony-uninformative characters (bp)	31	87	11	17	8
Parsimony-informative characters (bp)	25	50	16	26	7
Parsimony-uninformative characters (%)	4.29	6.40	1.52	1.25	1.49
Parsimony-informative characters (%)	3.46	3.68	2.21	1.91	1.30
Total % variability	7.75	10.07	3.73	3.16	2.79

### Phylogenetic analysis of the large-scale *Begonia* plastid alignment

The highly conservative nature of gene synteny in the plastid genome allowed the alignment of plastid contigs for all sixteen *Begonia* species at a genome-wide level. In this study, only one copy of the IR is included in the large-scale genome alignment because two copies of the inverted repeat could not be confirmed during gap-closure. Phylogenetic analysis was carried out on the large-scale genome alignment of sixteen incomplete plastid genome sequences to determine whether the uncertainty over species-level patterns within sect. *Gireoudia* revealed in Moonlight et al., (2015) could be resolved using plastid genome data [15].

**Fig 3:**
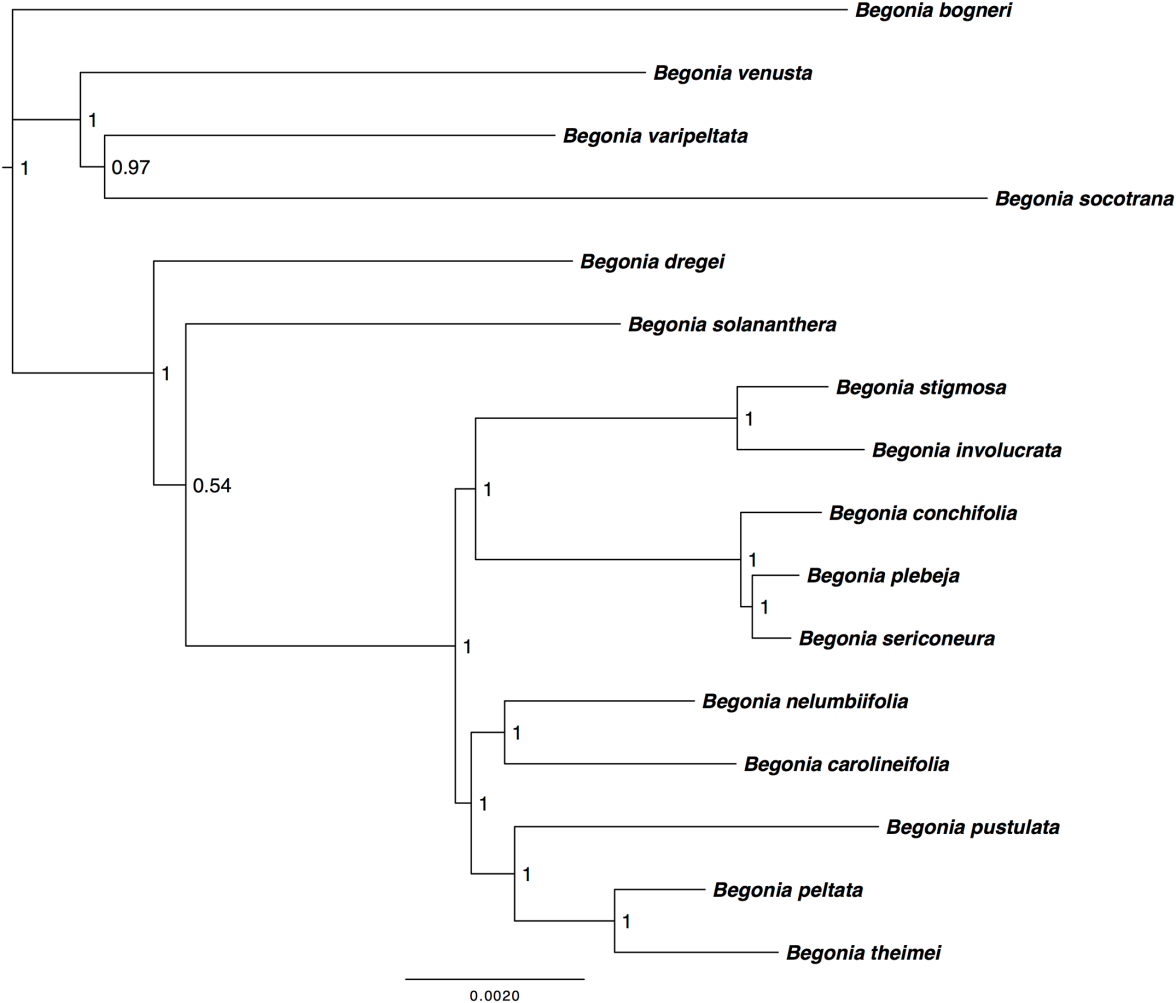
Bayesian Inference analysis of the large-scale *Begonia* plastid alignment. The large-scale *Begonia* plastid alignment has a length of 150,255 bp. The phylogentic tree depicts the evolutionary relationships between the sixteen *Begonia* species used in this study. Bayesian inference phylogenetic reconstruction was analysed under a GTR+I+G model performed in MrBayes with 16 taxa rooted on *B*. *bogneri*.

The aligned matrix for the large-scale *Begonia* plastid alignment consisted of 16 taxa with an alignment length of 150,255 bp, (139,457 bp unambiguously aligned characters, 143,538 characters are constant, 4804 variable parsimony-uninformative and 1,913 parsimony-informative sites). Phylogenetic analyses (MP, ML & BI) were performed on the large-scale *Begonia* alignment revealing sufficient phylogenetic variation was present to delineate the relationships of the sixteen *Begonia* species in this study (Fig 3). All three topologies were congruent with each other although there were minor differences between the levels of support and also the grouping of two taxa, namely *B*. *dregei* and *B*. *solananthera*. This uncertainty may be linked to evidence for at least two separate introductions of *Begonia* to the Neotropics and discordance between the mitochondrial, nuclear and plastid phylogenies at the base of the neotropical clades [17, 48].

### Phylogenetically informative plastid regions for low-level studies in *Begonia*

**Fig 4:**
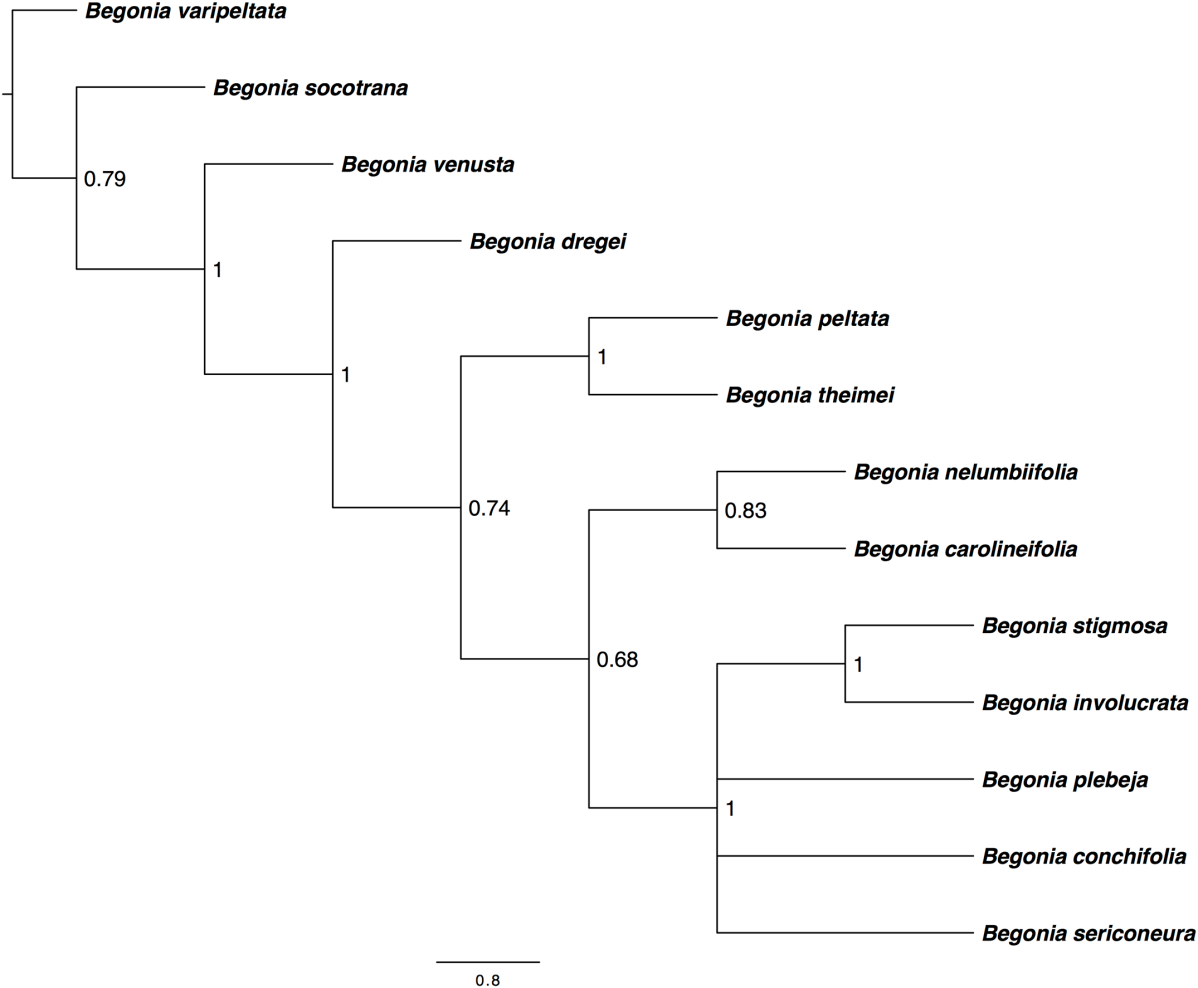
*Begonia* Phylogeny reconstruction using Region 21 (*ndhI-ndhG*) with an alignment length of 804 bp. Region 21 gave strong support within sect. *Gireoudia* along with good support outside of this group. Bayesian inference phylogenetic reconstruction analysed under a GTR+I+G model performed in MrBayes, with 13 taxa rooted on *B*.*varipeltata*.

**Fig 5:**
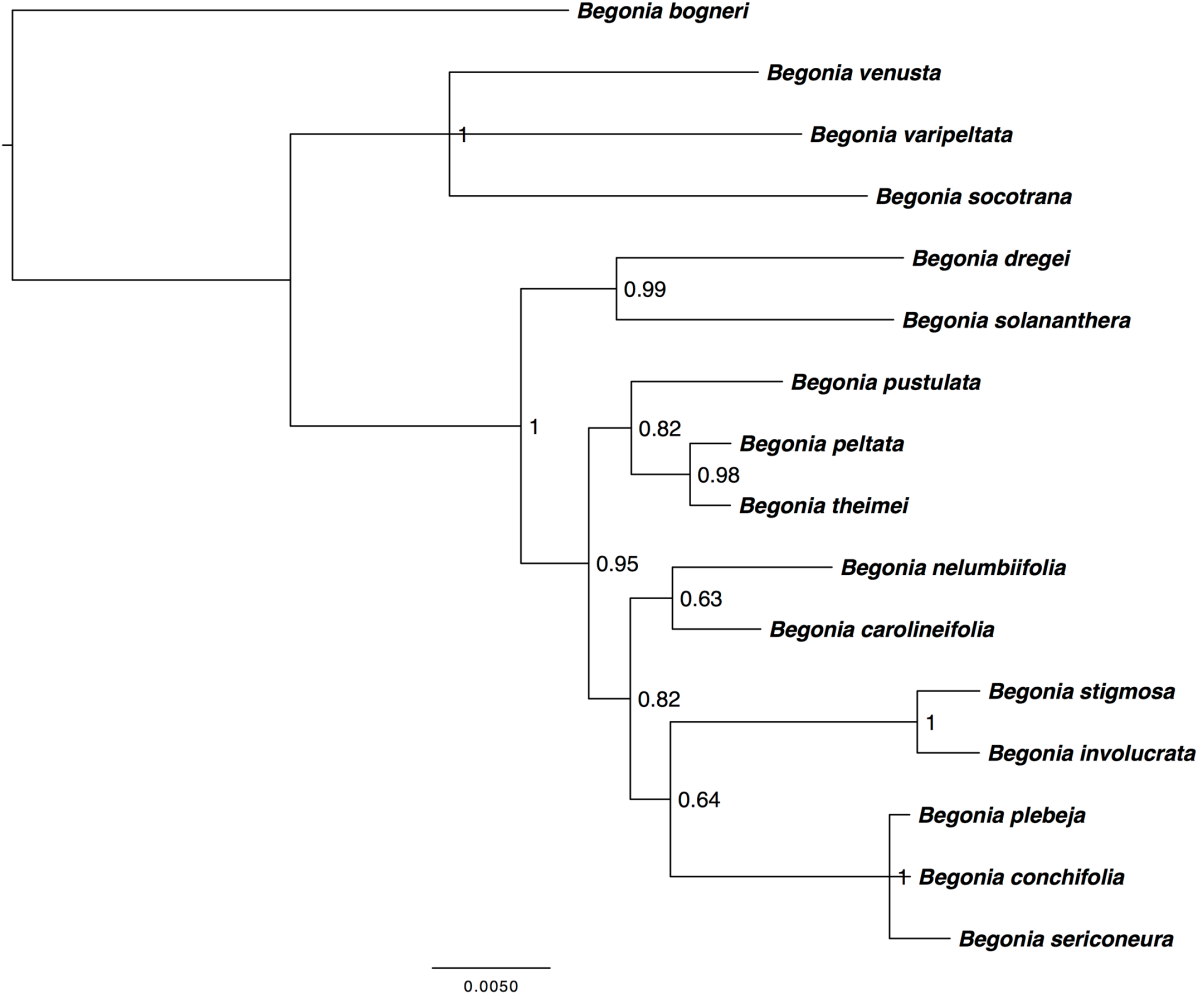
*Begonia* Phylogeny reconstruction using Region 24 (*rpoB*–*psbD*) with an alignment length of 1360 bp. Region 24 gave strong support within sect. *Gireoudia* along with good support outside of this group. Bayesian inference phylogenetic reconstruction analysed under a GTR+I+G model performed in MrBayes, with 16 taxa rooted on *B*. *bogneri*.

Twenty-four small regions (between 800–2000 bp in length) of the plastid genome alignment were chosen for phylogenetic analysis using Maximum likelihood analyses. The phylogenetic trees were assessed based on congruency with the results from the large-scale *Begonia* plastid alignment and tree node support values. Nine of the initial 24 regions were selected for further phylogenetic analyses using Bayesian Inference (Table 5). The majority of these plastid regions were part of the SSC, although one of the selected regions, Region 24, was in the LSC. Phylogenetic analyses determined that two regions, Region 21 and Region 24, contained high sequence divergence that could be suitable for phylogenetic analyses at the species level. These regions gave strong support within sect. *Gireoudia* along with good support outside of this group (Figs 4 and 5).

**Table 5:** A description of the nine phylogenetically informative small regions (between 1000–2000 bp in length) of the plastid genome alignment that were selected for advanced phylogenetic analyses using Bayesian Inference methods.

Region	Start Position (bp)	Stop Position (bp)	Dogma Annotation/Blast Match	Size of Alignment
3	39281	41175	Partial rpoC2 gene	1860 bp
4	41121	44766	Partial rpoC2 / rpoC1 genes	3640 bp
5	46915	49025	rpoB pseudogene	2111 bp
6	50026	51686	rpoB—trnC intergenic spacer	1330 bp (1661 bp with insertion)
7	52033	53183	trnC—trnD IG spacer. petN—psbM IG spacer	1151 bp
8	53175	54342	trnC—trnD IG spacer	1007 bp (1168 bp with insertion)
17	129364	130657	Partial ndhF gene	1294 bp
18	129364	131759	Partial ndhF gene	2370 bp
21	138658	139461	Partial ndhF gene	804 bp
24	49831	51190	rpoB—trnC intergenic spacer	1223 bp (1300 bp with insertion)

Region 21 (*ndhI-ndhG)* has an alignment length of 804 bp and is positioned on the large-scale plastid alignment at 138,658–139,461 bp spanning the intergenic region between the genes *ndhI* and *ndhG* in the SSC. An important feature of Region 21 is the relatively small size of the alignment meaning a full sequence is likely to be obtained from only one Sanger sequencing reaction, which is cost effective. It has an average pairwise identity of 86.7%, this is slightly higher when compared to an overall pairwise identity of the SSC alignment at 85.3%. Studies are now beginning to highlight the potential of non-coding regions within the SSC as putative sequences for phylogenetic analyses [47, 49].

Region 24 (*rpoB*–*psbD*) has an alignment length between 1223 bp (mostly sect. *Gireoudia* species) and 1300 bp (other *Begonia* species). It is located in the *rpo*B-*psb*M intergenic spacer in the LSC, (large-scale *Begonia* alignment position 27,734–28,931 bp) and has a higher pairwise identity than that of Region 21 at 93.9%. The pairwise identity is also much higher than the whole LSC (75.9%) however this is to be expected as there were large regions of missing data, especially for *B*. *pustulata*, *B solanathera* and *B*. *bogneri* and the real figure is likely to be much higher. Although, these analyses did not include indel-coding, it is speculated that Region 24 would be particularly suitable for the analysis of indels as there is a distinct indel structure in sect. *Gireoudia* that is not seen in the other *Begonia* sections. An area encompassing region 24 (*rpoB*–*psbD*, 28–36 Kb), has been reported by Wang and Messing, (2011) as a highly divergent region in the subfamily *Lemnoideae*, however their comparative plastid analysis was between genera, hence the resolution of a smaller region suitable for phylogenetic studies was not reported [50].

It is clear from the phylogenetic analyses that both regions give good support and resolution to the American *Begonia* species and also to the African and Asian species. These two regions, Region 21 (*ndhI-ndhG)* and Region 24 (*rpoB*–*psbD*) are recommended as candidates for further assessment in a wider sampling of the pantropical genus *Begonia*.

## Conclusions

The creation of this dataset provided better phylogenetic resolution of relationships within sect. *Gireoudia* and between closely related species, providing a strongly supported phylogenetic tree. The inclusion of plastid genomes from African and Asian *Begonia* species as outgroups, meant a comparative study could be undertaken, gaining new insights into the rates of evolution of different plastid regions and gene order. It also enabled the identification of appropriate informative loci for phylogenetic studies in *Begonia* at species level. The identification of two suitable molecular markers for the study of phylogenetics in *Begonia*, particularly Neotropical lineages means a comprehensive phylogenetic analysis can now be undertaken to fully understand how American *Begonia* have evolved throughout Mesoamerica.

In order to determine the evolutionary history of *Begonia*, it is necessary to understand the full genomic complement in *Begonia* species and further studies into nuclear and mitochondrial genome evolution are required. This study presents a framework for the discovery and development of new, and more importantly, suitable molecular markers for phylogenetic studies in *Begonia*. It is an important step in the development and selection of new, phylogenetically informative markers for non-model species for which little information is available.

## Supporting Information

S1 Table Primer sequences used in this manuscript to amplify plastid regions in *Begonia*. (DOCX)

S2 Table Custom primer sequences developed from *B*. *peltata plastid* genome to confirm the junctions of the inverted repeats (IR) and to close gaps between contigs. (DOCX)

S3 Table A total number of 32,611,570 sequence reads were generated, providing 6.2 Gb of data, consisting of 50bp paired-end illumina reads. Expected coverage for each accession was determined based on the size of the *Cucumis sativas* plastid genome.
(DOCX)
