# Supplementary material for: Comparative Analysis of Begonia Plastid Genomes and Their Utility for Species-Level Phylogenetics
Source: PLoS One. 2016 Apr 8;11(4):e0153248. doi: 10.1371/journal.pone.0153248 (PMC4825977; doi:10.1371/journal.pone.0153248)
Supplement: S1 Table — (DOCX) [file pone.0153248.s001.docx]

**S1 Table:** Primer sequences used in this manuscript to amplify plastid regions in *Begonia*.

| Primer Name | Position bp (*Nicotiana tabacum* chloroplast genome) | Strand | DNA Sequence (5'-3') |
| --- | --- | --- | --- |
| CC1F | 155747 | F | ayggrggtggtgaagggag |
| CC1R | 9533 | R | aacgaatcacacacttttaccacta |
| CC2F | 7918 | F | ggcctaygcytttttgaatcc |
| CC2R | 17040 | R | gagaaggttccatcggaatcc |
| CC3F | 16771 | F | tctgataaaaaacgagcagttct |
| CC3R | 24129 | R | yaaaccggaaaaagatggattatt |
| CC4F | 23085 | F | rggcctagcaaawgaaaaatt |
| CC4R | 31070 | R | ggcggtrtggataaaagawcttc |
| CC5F | 29455 | F | gcrccaktgattycactatta |
| CC5R | 37160 | R | gttcgaatacaccagctacac |
| CC6F | 36821 | F | cgggaagggctcgkgcag |
| CC6R | 46157 | R | cctattacagagatggtgygattt |
| CC7F | 45985 | F | gaggatacacgacagarggarttg |
| CC7R | 53882 | R | agttaatgaaagagcccaatgc |
| CC8F | 52505 | F | aagcatcsccsathggtt |
| CC8R | 62486 | R | aattgkttawatctaaaawaataacat |
| CC9F | 61113 | F | gccgaaccywaygccta |
| CC9R | 71700 | R | accatagaaacgawggaacccact |
| CC10F | 71536 | F | ttggctactctaaccttccc |
| CC10R | 81876 | R | accaaagggwgttattcatgttca |
| CC11F | 81248 | F | taktcrtgtggkaykttytcagat |
| CC11R | 90060 | R | ttggctagaatccgttacttgaa |
| CC12F | 89425 | F | tggaggaactggatcggaa |
| CC12R | 99798 | R | tcccgatatarcagtaaaagcaaga |
| CC13F | 99038 | F | agggcygttatgctcattacka |
| CC13R | 109862 | R | ggtgcgttccgrggtgtga |
| CC14F | 109627 | F | cacaccaatccatcccgaact |
| CC14R | 119284 | R | gctaarcaaatwgcttctgctcc |
| CC15F | 118974 | F | ccgcaratattggaaaaacwacaa |
| CC15R | 127904 | R | caagcatatgtttttacaaattatca |
| CC16F | 127696 | F | tgccgtcttaaccagttttt |
| CC16R | 136980 | R | gtagttggagtcggcggc |
| CC17F | 136271 | F | tccaccgtaagcctttcct |
| CC17R | 146859 | R | tggccatgaaakrgggattaa |
| CC18F | 146001 | F | atgtccracycgggcctatat |
| CC18R | 24 | R | tcaattcccgtcgttcgcc |
| CC2aF | 8488 | F | aatcctggacgtgaagaataa |
| CC2bF | 4281 | F | tttcaggatcagtcgtggtctt |
| CC2bR | 13967 | R | gcararggaaaaatacgaggt |
| CC2cF | 13302 | F | ycrcacacactccctttcc |
| CC2cR | 23689 | R | aaagggaattgatcyatggtcga |
| CC10aF | 71069 | F | tcrttmkaaatyrtataaakacaay |
| CC10aR | 80583 | R | ttggaaccttttgaataacagyc |
| CC11aF | 80193 | F | accgyrgysgccctktgg |
| CC11aR | 91987 | R | gaarcagaagtgatgtggattatt |
| CC6aF | 37137 | F | aaaaggagagagagggattcga |
| CC6aR | 49490 | R | cttccwttgagtctctgcacct |
