# Supplementary material for: Comparative Analysis of Begonia Plastid Genomes and Their Utility for Species-Level Phylogenetics
Source: PLoS One. 2016 Apr 8;11(4):e0153248. doi: 10.1371/journal.pone.0153248 (PMC4825977; doi:10.1371/journal.pone.0153248)
Supplement: S2 Table — (DOCX) [file pone.0153248.s002.docx]

**S2 Table:** Custom primer sequences developed from *B. peltata plastid* genome to confirm the junctions of the inverted repeats (IR) and to close gaps between contigs.

| **Primer Name** | **DNA Sequence 5' - 3'** |
| --- | --- |
| Node233.Fw | gttcgattcccgctacccgc |
| Node378.Rv | cttccaccaagacattcaccg |
| Node378.Fw | ccggctcaagtagttacatc |
| Node3.Rv | aggattagtatattgcgatg |
| Node3.Fw | ctgatatcttggcagcattcc |
| Node705.Rv | gattacgtggtacgattggatc |
| Node705.Fw | tcttcctagaaatggagagc |
| rpl2.Rv_1 | cttctaccccgagcacacgc |
| ndhF.Rv_1 | catatcaatattcgtggatcatc |
| atpB.Fw_1 | gcttcatcgatgttacctacc |
| rpl20.Fw | caagtattttacgattaagaag |
| trnR_ACG.Rv_1 | gcctgtagctcagaggattagagg |
| rps15.Fw_1 | actcccgaatatccaattgc |
| rps19_Rv | atagcaagagtatgaccaatc |
| rps19_Fw | gattggtcatactcttgctat |
| rpl16_Fw | gtacgtatatttccagacaaaccag |
| rpl16_Rv | ctggtttgtctggaaatatacgtac |
| atpB.Fw | ggtaggtaacatcgatgaagc |
| psa1.Rv | ggtaagttgttgaaagttgtc |
| petB.Rv | gcaatcgcctgaatttcaagacg |
| psbH.Fw | cctttgatgggtgttgcgatg |
| psbB.Rv | cggtatgaacacgataccaag |
| rpl20.Fw | cttcttaatcgtaaaatacttg |
| rps11.Fw | gttatatatgtgatgtaactcc |
| trnR_ACG.Rv | cctctaatcctctgagctacaggc |
| rps15.Fw | gcaattggatattcgggagt |
| trnI_GAU.Rv | ggcgcgctctaccactgagc |
| rpl2.Rv | gcgtgtgctcggggtagaag |
| trnI_GAU.Fw | gctcagtggtagagcgcgcc |
| ndhF.Rv | gatgatccacgaatattgatatg |
