# Supplementary material for: Comparative Analysis of Begonia Plastid Genomes and Their Utility for Species-Level Phylogenetics
Source: PLoS One. 2016 Apr 8;11(4):e0153248. doi: 10.1371/journal.pone.0153248 (PMC4825977; doi:10.1371/journal.pone.0153248)
Supplement: S3 Table — Expected coverage for each accession was determined based on the size of the Cucumis sativas plastid genome. (DOCX) [file pone.0153248.s003.docx]

**S3 Table:** A total number of 32,611,570 sequence reads were generated, providing 6.2 Gb of data, consisting of 50bp paired-end illumina reads. Expected coverage for each accession was determined based on the size of the *Cucumis sativas* plastid genome.

| *Begonia* Accession | Number of sequence reads | Expected Coverage based on *Cucumis sativus* plastid genome 155,277 bp |
| --- | --- | --- |
| *B. plebeja* | 1944684 | 626 |
| *B. conchifolia* | 1445356 | 465 |
| *B. stigmosa* | 2378254 | 766 |
| *B. peltata* | 2162762 | 696 |
| *B. nelumbiifolia* | 2405790 | 775 |
| *B. theimei* | 2198796 | 708 |
| *B. sericoneura* | 2341940 | 754 |
| *B. involucrata* | 2584350 | 832 |
| *B. pustulata* | 1187422 | 382 |
| *B. carolineifolia* | 2651204 | 854 |
| *B. solananthera* | 1675280 | 539 |
| *B. bogneri* | 1615962 | 520 |
| *B. venusta* | 1291502 | 416 |
| *B. varipeltata* | 2285542 | 736 |
| *B. dregei* | 2116728 | 682 |
| *B. socotrana* | 2325998 | 749 |
